# Prognostic value of patient-reported outcomes for survival in patients with advanced lung cancer receiving immune checkpoint inhibitors

**DOI:** 10.3389/fimmu.2025.1640595

**Published:** 2025-10-21

**Authors:** David Gandara, Miranda Gogishvili, Ahmet Sezer, Tamta Makharadze, Mahmut Gümüş, Cong Zhu, Eric Yan, Giuseppe Gullo, Petra Rietschel, Ruben G. W. Quek

**Affiliations:** ^1^ Division of Hematology/Oncology, Department of Medicine, UC Davis Comprehensive Cancer Center, Sacramento, CA, United States; ^2^ High Technology Medical Centre, University Clinic Ltd, Tbilisi, Georgia; ^3^ Department of Medical Oncology, Başkent University, Adana, Türkiye; ^4^ LTD High Technology Hospital Med Center, Batumi, Georgia; ^5^ Department of Medical Oncology, School of Medicine, Istanbul Medeniyet University, Istanbul, Türkiye; ^6^ Regeneron Pharmaceuticals, Inc., Tarrytown, NY, United States; ^7^ Cyan Global Inc., San Diego, CA, United States

**Keywords:** non-small cell lung cancer, immunotherapy, patient-reported outcomes, immune checkpoint inhibitors, cemiplimab, quality of life

## Abstract

**Introduction:**

There is potential clinical utility in using patient-reported outcomes (PROs) to predict survival in patients with advanced non-small cell lung cancer. We assessed the prognostic value of PROs for survival in two phase 3 cemiplimab studies in advanced non-small cell lung cancer.

**Methods:**

Data from EMPOWER-Lung 1 and EMPOWER-Lung 3 Part 2, two global, randomized phase 3 clinical trials, were used. Patients with advanced non-small cell lung cancer and programmed cell death-ligand 1 expression ≥50% received cemiplimab monotherapy (n=283), and patients with no *EGFR*, *ALK*, or *ROS1* genomic aberrations received cemiplimab plus chemotherapy (n=312). PROs were assessed using the European Organization for Research and Treatment of Cancer Core Quality of Life and Quality of Life Lung Cancer 13 questionnaires. Association between baseline PROs and survival was analyzed, and the C-statistic was used to assess the prognostic value of PROs in comparison with the Eastern Cooperative Oncology Group performance status (ECOG PS) scale.

**Results:**

Twenty-five PROs were evaluated, of which 15 were significantly associated (*P*<0.05) with overall survival and were better predictors than ECOG PS. Fourteen PROs were significantly associated (*P*<0.05) with progression-free survival; of these, 13 had better prognostic value than ECOG PS. Patient-reported dyspnea and physical functioning had the highest prognostic values for overall survival (c=0.635 and c=0.619, respectively) and progression-free survival (c=0.593 and c=0.583, respectively). Stratifying physical functioning into high, medium, and low categories showed that patients with high physical functioning at baseline had significantly better overall survival (high vs low; HR, 0.41; 95% CI, 0.23-0.71; *P*=0.001), resulting in a 59% reduction in the risk of death. Similarly, patients in the high physical functioning category had significantly favorable progression-free survival (high vs low; HR, 0.44, 95% CI, 0.29-0.66; *P*<0.001) and a 56% reduction in the risk of death.

**Conclusion:**

Baseline PROs, including dyspnea and physical functioning, have significant prognostic value for survival for patients with advanced non-small cell lung cancer.

## 1 Introduction

The therapeutic landscape in advanced non-small-cell lung cancer (NSCLC) has changed rapidly in recent years. For the majority of patients with non−oncogene-driven cancers, immune checkpoint inhibitor (ICI) therapy has revolutionized therapeutic decision-making. ICI therapeutic options vary widely; these include anti-programmed cell death-1 (PD-1)/programmed cell death-ligand 1 (PD-L1) agent monotherapy, combination therapy with radiotherapy or chemotherapy, and immunotherapy combinations (anti–PD-1/PD-L1 plus anti−cytotoxic T-lymphocyte antigen 4) (1–4).

Cemiplimab (a PD-1 inhibitor) is approved internationally as a first-line treatment for patients with advanced NSCLC with a PD-L1 expression level of ≥50%, as well as in combination with platinum-based chemotherapy for patients with advanced NSCLC with no epidermal growth factor receptor (*EGFR*), anaplastic lymphoma kinase (*ALK*), or c-ros oncogene 1 (*ROS1*) genomic aberrations (5–8). The approval of cemiplimab was based on published data from two pivotal phase 3 trials, EMPOWER-Lung 1 (NCT03088540) and EMPOWER-Lung 3 Part 2 (NCT03409614) (**Supplementary Table S1**) (9–13). Results from these studies showed clinically meaningful and statistically significant improvement in overall survival (OS) and progression-free survival (PFS) with first-line cemiplimab ± chemotherapy compared with platinum-doublet chemotherapy in patients with advanced NSCLC (9, 13). The safety results of cemiplimab in EMPOWER-Lung 1 and EMPOWER-Lung 3 were generally consistent with that of other immunotherapy-based trials in first-line treatment of advanced NSCLC (9, 13).

Patient-reported outcomes (PROs) in advanced NSCLC have previously also been examined for first-line cemiplimab (as monotherapy and in combination with chemotherapy) in EMPOWER Lung 1 and EMPOWER Lung 3 (14, 15); significant overall improvement in symptoms and delayed time to definitive clinically meaningful deterioration in cancer-related and lung cancer–specific symptoms and functions were observed.

Most recently, PROs have shown promise in their prognostic value for survival (16); however, no studies have assessed the prognostic performance of PROs in patients with advanced NSCLC initiating first-line cemiplimab-based therapy. Quality-of-life data have also been underutilized in a significant number of phase 3 lung cancer trials (17, 18). This study therefore aimed to evaluate the prognostic value of PROs for survival in patients from the two pivotal phase 3 cemiplimab trials, EMPOWER-Lung 1 and EMPOWER-Lung 3, in advanced NSCLC.

The physician-reported Eastern Cooperative Oncology Group performance status (ECOG PS) scale is influenced by several factors, such as the burden of the disease itself, the presence of comorbidities, and global frailty of elderly people (16), and relying on ECOG PS alone as a prognostic clinical marker may be insufficient. Patient-reported physical function has been shown to be a tumor-agnostic predictor of OS, which in several analyses has been more prognostic than physician-assessed ECOG PS (19–24). This study therefore aimed to further evaluate the prognostic value of patient-reported risk-stratified physical functioning in patients with advanced NSCLC initiating first-line cemiplimab-based therapy.

## 2 Methods

### 2.1 Inclusion criteria and trial description

The study designs have been described previously (9, 10). Briefly, patients with advanced NSCLC were included in the cemiplimab monotherapy and cemiplimab plus chemotherapy treatment arms for the EMPOWER-Lung 1 and EMPOWER-Lung 3 Part 2 phase 3 clinical trials. Both trials included patients aged ≥18 years with squamous and non-squamous advanced NSCLC; however, EMPOWER-Lung 1 included patients with PD-L1 expression ≥50% and EMPOWER-Lung 3 Part 2 included patients with no *EGFR*, *ALK*, or *ROS1* genomic aberrations (9, 10).

### 2.2 Predictor and patient-reported outcome measures

The physician-defined ECOG PS scale is widely used to assess the functional status of patients with cancer, including their ability to self-care, ability to perform daily activities, physical ability (ie, walking and working), and ability to tolerate treatment. ECOG PS scores range from 0 (fully active) to 5 (dead); higher scores represent poorer functioning (25).

PROs were assessed using the European Organization for Research and Treatment of Cancer (EORTC) Core Quality of Life (QLQ-C30) and Quality of Life Lung Cancer 13 (QLQ-LC13) questionnaires. The QLQ-C30 questionnaire assesses functioning (physical, role, cognitive, emotional, and social), symptoms (fatigue, pain, nausea, vomiting, dyspnea, appetite loss, insomnia, constipation, and diarrhea), global health, and quality of life (26). As a supplementary module to the QLQ-C30, the QLQ-LC13 questionnaire measures lung-cancer–associated symptoms (coughing, hemoptysis, dyspnea, and pain) and side effects from conventional chemotherapy and radiotherapy (hair loss, neuropathy, sore mouth, and dysphagia) (27).

The EORTC QLQ-C30 and QLQ-LC13 questionnaires were scored per the instrument scoring manual (28). For both questionnaires, scores range from 0 to 100; higher scores on functioning and global health/quality of life scales indicate better outcomes, and higher scores on symptom scales indicate worse outcomes, from a patient perspective (26, 27, 29–31).

### 2.3 Statistical analysis

Individual patient data from cemiplimab-based therapy in the EMPOWER-Lung 1 and EMPOWER-Lung 3 Part 2 studies were utilized to evaluate the association between baseline PROs and survival by Cox proportional hazard regression, stratified by treatment, histology, and PD-L1 level. Statistically significant results were reported with HRs and 95% CIs, and statistical significance was pre-defined at *P*<0.05. HRs were based on a 10-point increase in the EORTC QLQ-C30 and QLQ-LC13 scales.

The prediction performance of PROs was assessed using Harrell’s concordance statistic, or C-statistic, which measures the concordance between PROs and OS (32). The higher the C-statistic, the better the model predicts OS outcomes (33).

For patient-reported physical functioning, the prognostic performance was also compared against the ECOG PS scale. The association between survival outcomes and baseline physical functioning was assessed by Kaplan–Meier analysis (stratified by low, intermediate, and high categories per EORTC QLQ-C30 Lung Cancer Stage III/IV (34)–specified interquartile definitions with thresholds being: low, <46.7; intermediate, ≥46.7–≤86.7; and high, >86.7).

C-statistic computations were performed using R programming language (GNU Project); SAS version 9.4 (SAS Institute Inc) was used for all other statistical analyses.

### 2.4 Ethical consideration

The study was conducted in accordance with the Declaration of Helsinki (as revised in 2013) and the International Conference on Harmonization Good Clinical Practice guidelines. All patients provided written informed consent (9, 10).

## 3 Results

### 3.1 Patient population

In the pooled cohort, 283 patients with advanced NSCLC and PD-L1 expression ≥50% were treated with cemiplimab monotherapy, and 312 patients with advanced NSCLC and no *EGFR*, *ALK*, or *ROS1* genomic aberrations were treated with cemiplimab plus chemotherapy (9, 10). **Supplementary Table S2** summarizes the baseline patient characteristics by treatment arms, which were broadly similar. For every cycle from baseline to Cycle 27, ≥90% of patients who received cemiplimab monotherapy and ≥90% of patients who received cemiplimab plus chemotherapy completed at least one question on each of the EORTC QLQ-C30 and QLQ-LC13 questionnaires.

### 3.2 Patient-reported outcomes

Out of the 25 PROs analyzed, 15 were significantly associated with OS (*P*<0.05) and had greater prognostic value than physician-reported ECOG PS. Patient-reported dyspnea (per EORTC QLQ-LC13; c=0.635) and physical functioning (per EORTC QLQ-C30; c=0.619) had the highest predictability for OS (**Table 1**).

**Table 1:** Rank summary of the prognostic value of PROs for OS for patients in the overall population.

Variables	N	HR^a^ (95% CI)	*P*-value	C-statistic
EORTC QLQ-C30 and QLQ-LC13 scales				
Continuous variables				
LC-dyspnea	591	1.19 (1.12–1.26)	<0.001	0.635
Physical functioning	592	0.84 (0.78–0.90)	<0.001	0.619
Fatigue	592	1.15 (1.08–1.23)	<0.001	0.601
Role functioning	592	0.90 (0.85–0.94)	<0.001	0.600
Dyspnea	592	1.11 (1.05–1.16)	<0.001	0.598
Social functioning	593	0.88 (0.83–0.93)	<0.001	0.597
Financial problems	591	1.08 (1.03–1.13)	0.001	0.587
Pain	593	1.11 (1.05–1.17)	<0.001	0.586
LC-pain in other parts	590	1.08 (1.03–1.14)	0.003	0.572
Insomnia	592	1.07 (1.02–1.12)	0.004	0.568
GHS/QoL	593	0.91 (0.85–0.98)	0.012	0.564
Constipation	593	1.08 (1.02–1.14)	0.006	0.550
Appetite loss	592	1.08 (1.02–1.13)	0.006	0.548
Emotional functioning	593	0.93 (0.87–1.00)	0.048	0.545
LC-coughing	592	1.06 (1.0–1.11)	0.050	0.540
LC-dysphagia	593	1.09 (1.02–1.17)	0.017	0.539
Nausea/vomiting	592	1.07 (0.98–1.17)	0.135	0.530
LC-pain in chest	593	1.04 (0.99–1.10)	0.138	0.530
LC-pain in arm or shoulder	592	1.05 (1.00–1.11)	0.060	0.520
Diarrhea	592	0.97 (0.87–1.08)	0.551	0.518
Cognitive functioning	593	1.00 (0.92–1.09)	0.977	0.514
LC-sore mouth	593	1.00 (0.90–1.12)	0.959	0.499
LC-peripheral neuropathy	592	0.97 (0.89–1.06)	0.562	0.498
LC-alopecia	593	1.03 (0.95–1.11)	0.499	0.497
LC-hemoptysis	592	0.97 (0.87–1.08)	0.549	0.483
Categorical variables				
Physical functioning	592			0.571
Intermediate vs low		0.71 (0.46–1.08)	0.107	
High vs low		0.41 (0.23–0.71)	0.001	
**ECOG PS**	595	1.38 (0.94–2.05)	0.104	0.534

CI, confidence interval; ECOG PS, Eastern Cooperative Oncology Group performance status; EORTC, European Organization for Research and Treatment of Cancer; GHS, global health status; HR, hazard ratio; LC, lung cancer; OS, overall survival; PRO, patient-reported outcome; QLQ-C30, Core Quality of Life; QLQ-LC13, Quality of Life Lung Cancer 13; QoL, quality of life.

ECOG PS was analyzed as a categorical variable.

a HR is based on a 10-point increase in the EORTC QLQ-C30/LC13 scales.

Fourteen out of 25 PROs were significantly associated with PFS (*P*<0.05), and 13 of the statistically significant PROs had higher prognostic performance than the ECOG PS. For PFS, patient-reported dyspnea (per EORTC QLQ-LC13; c=0.593) and physical functioning (per EORTC QLQ-C30; c=0.583) had the highest predictability (**Table 2**).

**Table 2:** Rank summary of the prognostic value of PROs for PFS for patients in the overall population.

Variables	N	HR^a^ (95% CI)	*P*-value	C-statistic
EORTC QLQ-C30 and QLQ-LC13 scales				
Continuous variables				
LC-dyspnea	591	1.14 (1.09–1.20)	<0.001	0.593
Physical functioning	592	0.86 (0.81–0.91)	<0.001	0.583
Social functioning	593	0.90 (0.86–0.94)	<0.001	0.579
Role functioning	592	0.91 (0.87–0.94)	<0.001	0.573
Fatigue	592	1.13 (1.08–1.19)	<0.001	0.572
Dyspnea	592	1.09 (1.05–1.13)	<0.001	0.568
GHS/QoL	593	0.92 (0.87–0.97)	0.001	0.554
Financial problems	591	1.04 (1.01–1.08)	0.017	0.548
Pain	593	1.07 (1.03–1.12)	0.002	0.544
Emotional functioning	593	0.93 (0.88–0.98)	0.011	0.544
Insomnia	592	1.05 (1.01–1.09)	0.015	0.542
Appetite loss	592	1.07 (1.02–1.11)	0.002	0.532
Constipation	593	1.07 (1.03–1.12)	0.002	0.530
LC-coughing	592	1.04 (0.99–1.08)	0.095	0.530
LC-pain in other parts	590	1.05 (1.01–1.09)	0.022	0.529
LC-dysphagia	593	1.06 (1.00–1.13)	0.042	0.521
Nausea/vomiting	592	1.04 (0.97–1.12)	0.300	0.519
LC-pain in chest	593	1.03 (0.99–1.07)	0.123	0.518
LC-pain in arm or shoulder	592	1.03 (0.99–1.08)	0.121	0.512
LC-peripheral neuropathy	592	1.00 (0.94–1.08)	0.879	0.504
LC-sore mouth	593	1.03 (0.96–1.11)	0.434	0.502
Cognitive functioning	593	1.00 (0.93–1.06)	0.875	0.502
Diarrhea	592	1.03 (0.96–1.11)	0.446	0.498
LC-hemoptysis	592	0.99 (0.92–1.07)	0.778	0.492
LC-alopecia	593	1.00 (0.93–1.08)	0.916	0.491
Categorical variables				
Physical functioning	592			0.551
Intermediate vs low		0.66 (0.47–0.92)	0.015	
High vs low		0.44 (0.29–0.66)	<0.001	
**ECOG PS**	595	1.36	0.037	0.527

CI, confidence interval; ECOG PS, Eastern Cooperative Oncology Group performance status; EORTC, European Organization for Research and Treatment of Cancer; GHS, global health status; HR, hazard ratio; LC, lung cancer; PFS, progression-free survival; PRO, patient-reported outcome; QLQ-C30, Core Quality of Life; QLQ-LC13, Quality of Life Lung Cancer 13; QoL, quality of life.

ECOG PS was analyzed as a categorical variable.

a HR is based on a 10-point increase in the EORTC QLQ-C30/LC13 scales.

**Figure 1:**
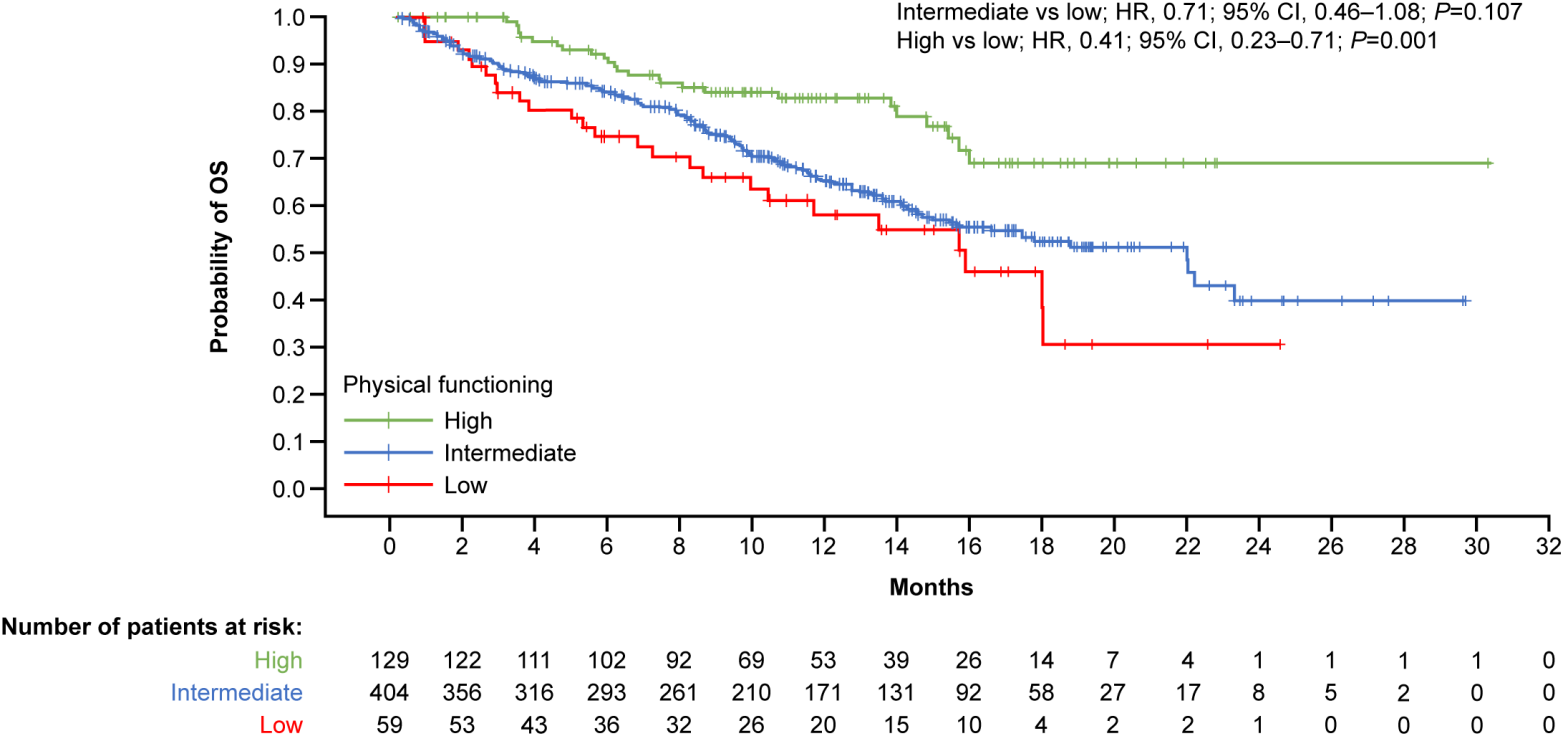
Kaplan–Meier curves by physical functioning at baseline for OS. Physical functioning baseline scores per EORTC QLQ-C30 Lung Cancer Model – Stage III/IV interquartile categories: low, <46.7; intermediate, ≥46.7–≤86.7; high, >86.7. CI, confidence interval; EORTC, European Organization for Research and Treatment of Cancer; HR, hazard ratio; OS, overall survival; QLQ-C30, Core Quality of Life.

When baseline physical functioning was stratified by low, intermediate, and high categories, results from the survival analyses showed that patients with high baseline physical functioning had significantly more favorable OS than those with low physical functioning (high vs low; HR, 0.41; 95% CI, 0.23–0.71; *P*=0.001) (**Figure 1**), representing a predicted 59% reduction in the risk of death.

**Figure 2:**
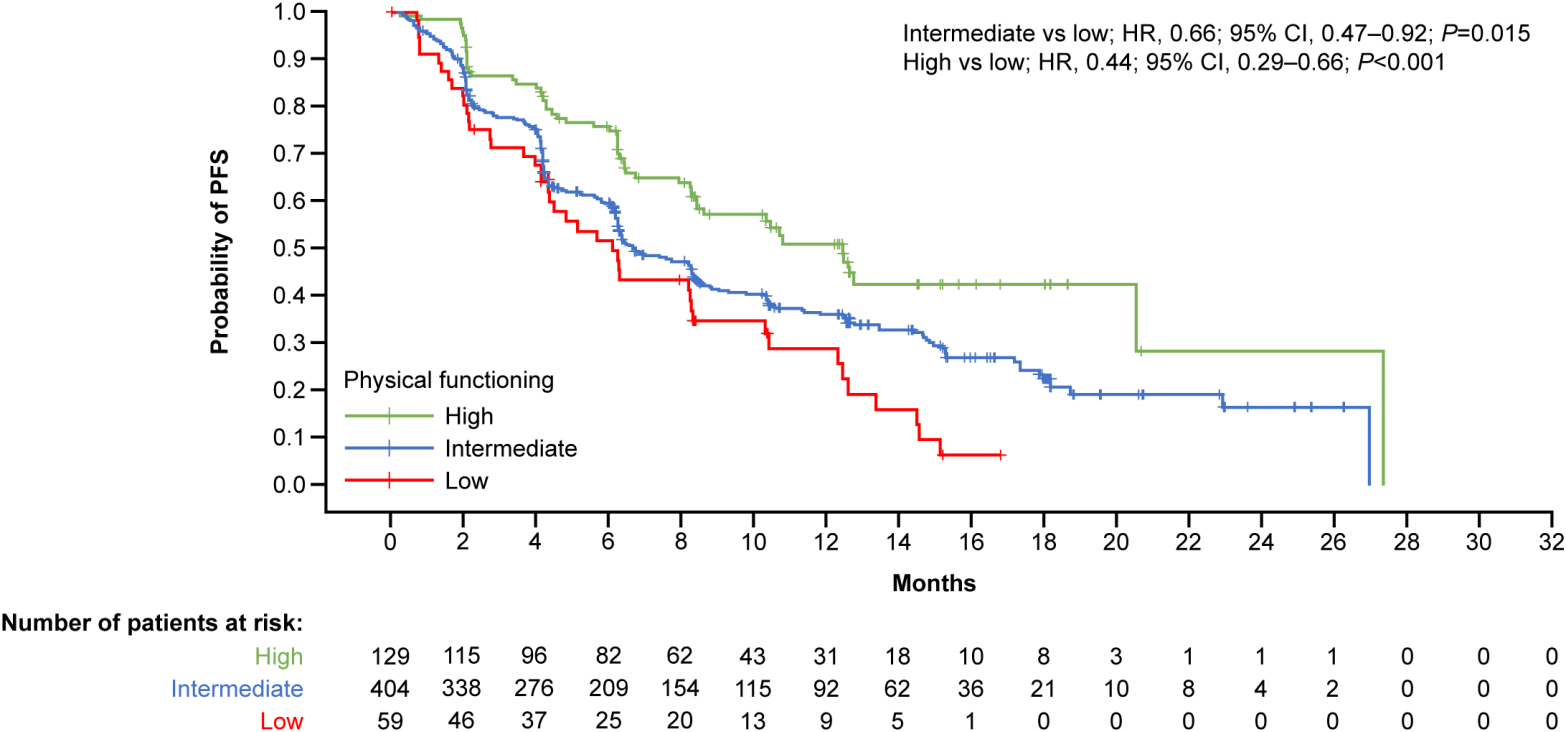
Kaplan–Meier curves by physical functioning at baseline for PFS. Physical functioning baseline scores per EORTC QLQ-C30 Lung Cancer Module – stage III/IV interquartile categories: low, <46.7; intermediate, ≥46.7–≤86.7; high, >86.7. CI, confidence interval; EORTC, European Organization for Research and Treatment of Cancer; HR, hazard ratio; PFS, progression-free survival; QLQ-C30, Core Quality of Life.

Likewise, patients with high baseline physical functioning had significantly more favorable PFS than those with low physical functioning (high vs low; HR, 0.44; 95% CI, 0.29-0.66; *P*<0.001) (**Figure 2**), representing a predicted 56% reduction in the risk of death.

## 4 Discussion

While recent studies have increasingly incorporated PROs into cancer clinical trial design and interpretation, there remains wide variability in the actual measures and analytic approaches employed (35). Most importantly, studies have found discordance between physician and patient perspectives, including underreporting of toxicities by physicians (36, 37). Further, studies of ECOG PS have shown that 40–50% of physicians overestimate patients’ performance status (36, 38). In our study, 15 and 14 out of 25 baseline PROs had better prognostic performance for OS and PFS, respectively, than physician-defined ECOG PS (0–1) in patients with advanced NSCLC initiating first-line cemiplimab-based therapy. The two baseline PROs with the highest predictability for OS and PFS were patient-reported dyspnea and physical functioning. Stratification at baseline for high versus low physical functioning categories revealed significant separation between patients, creating low-risk and high-risk patient groups.

The US Food and Drug Administration and other regulatory bodies have acknowledged the added value of incorporating PRO symptom and functional scales into clinical trial assessments. A set of key PROs that may contribute to a patient’s health-related quality of life and may provide a more sensitive measure of the effects of disease and treatment has been previously explored (39). More recently, the US Food and Drug Administration expanded on this concept by publishing guidance recommending the collection of specific core PROs in cancer clinical trials; these include disease-related symptoms, physical function, and role function (40).

In our analysis, PROs with functioning scales (e.g. physical) and select disease-related symptom scales (e.g. dyspnea) showed better prognostic performance for OS and PFS than ECOG PS. These may help clinicians prioritize select PROs with the most meaningful and measurable outcomes that predict OS and PFS in advanced NSCLC, thereby minimizing the burden on the patient and increasing the quality of collected data. This could help streamline PRO assessments in clinical trials and increase the regulatory utility of PRO data.

Furthermore, the prognostic value of PROs for survival could be useful in an increasingly patient-centered clinical setting. For example, the Enhancing Oncology model requires participating practices to collect and monitor PRO data (41). Such tools can increase patients’ self-awareness of symptoms and involvement in their care, better identify patient needs, inform treatment decisions, and improve cancer outcomes (41). The Centers for Medicare & Medicaid Services have outlined several domains for inclusion in PRO surveys, including symptom, functioning, and behavioral scales, such as the EORTC quality of life questionnaires (41).

Larger-scale studies designed to assess the prognostic value of PROs are needed to confirm these results. This analysis only included data from patients with advanced NSCLC who received first-line cemiplimab-based treatment and results may not be generalizable to other tumor and treatment types; future research should explore the validity of our results among patients receiving other therapies. Our study included patients from open-label trials (EMPOWER-Lung 1 and EMPOWER-Lung 3 Part 2), and the PRO results presented may be subject to patient biases. Of note, there is a lack of clear empirical evidence that such biases are sufficient to meaningfully affect the results of clinical trials (42). PROs collected from patients with advanced NSCLC in a real-world setting would be warranted to validate these results; especially among patients with ECOG PS >1 as our research is limited to patients with ECOG PS 0–1. Future analyses should explore interaction analyses and sensitivity analyses such as imputation methods to assess effects on the research outcomes. Future studies in a real-world setting that include patients with ECOG PS >1 (e.g. the CEMI-LUNG observational study [NCT05363319]) (43) should also be explored to evaluate the generalizability of our results.

## 5 Conclusions

In patients with advanced NSCLC who received first line cemiplimab based therapy, baseline PROs such as dyspnea and physical functioning have clinical utility in predicting patient survival in advanced NSCLC. These results suggest PROs have significant worth in oncology clinical practice and research trials of ICIs.

## Data Availability

The datasets presented in this article are not readily available because data will be made available by request. Qualified researchers may request access to study documents (including the clinical study report, study protocol with any amendments, blank case report form and statistical analysis plan) that support the methods and findings reported in this article. Individual anonymized participant data will be considered for sharing once the product and indication has been approved by major health authorities (eg, US Food and Drug Administration, European Medicines Agency, Pharmaceuticals and Medical Devices Agency, etc.), if there is legal authority to share the data and there is not a reasonable likelihood of participant re-identification. Requests to access the datasets should be directed to https://vivli.org/.
