# Supplementary material for: Prognostic value of patient-reported outcomes for survival in patients with advanced lung cancer receiving immune checkpoint inhibitors
Source: Front Immunol. 2025 Oct 21;16:1640595. doi: 10.3389/fimmu.2025.1640595 (PMC12583077; doi:10.3389/fimmu.2025.1640595)
Supplement: Supplementary file 1 [file Table1.docx]

Supplementary Material

**eTable 1. Study Designs for EMPOWER-Lung 1 and EMPOWER-Lung 3 Part 2**

|  | EMPOWER-Lung 1^1^ | EMPOWER-Lung 3 Part 2^2^ |
| --- | --- | --- |
| **NCT identifier** | NCT03088540 | NCT03409614 |
| **Patient population** | Advanced or metastatic NSCLC with PD-L1 expression ≥50% | Advanced or metastatic NSCLC and any level of PD-L1 expression |
| **Treatment arms** | 1:1 randomization  **Arm A**  Patients received cemiplimab 350 mg IV Q3W for 108 weeks or until disease progression  After disease progression, patients were given the option to continue cemiplimab + 4 cycles of chemotherapy  **Arm B**  Patients received 4-6 cycles of investigator’s choice of chemotherapy  After disease progression, patients were given the option to crossover to cemiplimab monotherapy | 2:1 randomization  **Arm A**  Patients received cemiplimab 350 mg IV Q3W + investigator’s choice of platinum doublet chemotherapy for 4 cycles^†^  **Arm B**  Patients received placebo Q3W + investigator’s choice of platinum doublet chemotherapy for 4 cycles^a^ |

IV, intravenous; NSCLC, non-small cell lung cancer; PD-L1, programmed cell death-ligand 1; Q3W, every 3 weeks.

^a^From randomization to data cutoff date.

# References

1. Sezer A, Kilickap S, Gümüş M, Bondarenko I, Özgüroğlu M, Gogishvili M, et al. Cemiplimab monotherapy for first-line treatment of advanced non-small-cell lung cancer with PD-L1 of at least 50%: A multicentre, open-label, global, phase 3, randomised, controlled trial. *Lancet*. (2021) 397:592-604. doi:10.1016/s0140-6736(21)00228-2.
2. Makharadze T, Gogishvili M, Melkadze T, Baramidze A, Giorgadze D, Penkov K, et al. Cemiplimab plus chemotherapy versus chemotherapy alone in advanced NSCLC: 2-year follow-up from the phase 3 EMPOWER-Lung 3 Part 2 trial. *J Thorac Oncol*. (2023) 18:755-768. doi:10.1016/j.jtho.2023.03.008.

**eTable 2. Baseline characteristics of patients in EMPOWER-Lung 1 and EMPOWER-Lung 3 Part 2**

|  | **Cemiplimab (n=283)** | **Cemiplimab + chemotherapy (n=312)** | **Overall (N=595)** |
| --- | --- | --- | --- |
| Age, years, median (IQR) | 63 (58–69) | 63 (57–68) | 63 (57–68) |
| <65, n (%) | 157 (55.5) | 184 (59.0) | 341 (57.3) |
| ≥65, n (%) | 126 (44.5) | 128 (41.0) | 254 (42.7) |
| Sex, n (%) |  |  |  |
| Male | 248 (87.6) | 268 (85.9) | 516 (86.7) |
| Female | 35 (12.4) | 44 (14.1) | 79 (13.3) |
| Race, n (%) | | | |
| White | 243 (85.9) | 267 (85.6) | 510 (85.7) |
| Black or African American | 1 (0.4) | 0 | 1 (0.2) |
| Asian | 31 (11.0) | 45 (14.4) | 76 (12.8) |
| American Indian or Alaska Native | 6 (2.1) | 0 | 6 (1.0) |
| Other | 2 (0.7) | 0 | 2 (0.3) |
| Ethnicity, n (%) | | | |
| Not Hispanic or Latino | 251 (88.7) | 311 (99.7) | 562 (94.5) |
| Hispanic or Latino | 32 (11.3) | 0 | 32 (5.4) |
| Not reported | 0 | 1 (0.3) | 1 (0.2) |
| Geographic region, n (%) | | | |
| Europe | 215 (76.0) | 270 (86.5) | 485 (81.5) |
| Asia | 31 (11.0) | 42 (13.5) | 73 (12.3) |
| Rest of the world | 37 (13.1) | 0 | 37 (6.2) |
| BMI, kg/m^2^, median (IQR) | 24 (21–27) | 25 (21–28) | 24 (21–28) |
| ECOG PS, n (%) | | | |
| 0 | 77 (27.2) | 51 (16.3) | 128 (21.5) |
| 1 | 206 (72.8) | 259 (83.0) | 465 (78.2) |
| Missing | 0 | 2 (0.6) | 2 (0.3) |
| Smoking status, n (%) | | | |
| Current | 105 (37.1) | 173 (55.4) | 278 (46.7) |
| Past | 178 (62.9) | 96 (30.8) | 274 (46.1) |
| Never | 0 | 43 (13.8) | 43 (7.2) |

BMI, body mass index; ECOG PS, Eastern Cooperative Oncology Group performance status; IQR, interquartile range.
